# Physiological and molecular processes of plant tolerance to bicarbonate-induced alkaline stress

**DOI:** 10.1007/s44154-026-00325-1

**Published:** 2026-07-27

**Authors:** Jiahe Fu, Liangliang Li, Minghui Xing, Wei Xie, Chenbo Zhu, Kaixin Yang, Xiaofan Nie, Xiaojian Yin, Mohammad Golam Mostofa, David Burritt, Lam-Son Phan Tran, Yuanyuan Bu, Weiqiang Li

**Affiliations:** 1 Key Laboratory of Saline-Alkali Vegetation Ecology Restoration, Ministry of Education, College of Life Sciences, Northeast Forestry University, Harbin, Heilongjiang, 150040 China; 2 Jilin Da’an Agro-Ecosystem National Observation and Research Station, State Key Laboratory of Black Soils Conservation and Utilization, Northeast Institute of Geography and Agroecology, Chinese Academy of Sciences, Changchun, 130102 China; 3 Department of Chemistry, State University of New York College of Environmental Science and Forestry, Syracuse, NY USA; 4 Department of Botany, The University of Otago, Dunedin, 9016 New Zealand; 5 Institute of Genomics for Crop Abiotic Stress Tolerance, Department of Plant and Soil Science, Texas Tech University, Lubbock, TX 79409 USA

**Keywords:** Alkaline stress, Bicarbonate, Halophytes, Ion homeostasis, Organic acid, Rhizosphere interactions

## Abstract

Bicarbonate-dominated alkaline soils represent a major class of salt-affected soils that can severely limit plant growth due to the combined effect of ionic toxicity, osmotic stress, and high pH. These soils exist in many parts of the world and often limit crop productivity due to the so-called alkaline stress in the rhizosphere. Alkaline stress reduces bioavailability of essential nutrients required for plant growth, such as iron, phosphates, and manganese, disrupts proton homeostasis, and perturbs cellular redox balance. Elucidating the mechanisms that enable plants to adapt to and grow in alkaline soils is essential if saline-alkali agriculture, where crops are grown on soils with high salt concentrations and high pH, is to contribute to global food security. Here, we summarize recent advances in the physiological, biochemical, and molecular processes that enable plants to tolerate and grow in alkaline soils. Key physiological mechanisms reviewed include plasma membrane H⁺-ATPase-driven proton extrusion, regulation of ion transporters, secretion of organic acids to acidify the rhizosphere, vacuolar sequestration of sodium and associated anions, and accumulation of compatible solutes for osmotic adjustment. At the molecular level, we also review and evaluate the genes, signaling pathways, and transcription factor networks underlying alkaline stress tolerance, with an emphasis on modules governing ion/redox homeostasis and root development. We also summarize emerging evidence on root-microbe interactions and their contributions to enhanced alkaline tolerance in plants. Finally, we discuss key requirements and future research directions for translating alkaline-tolerance mechanisms into crop improvement for alkaline soils.

## Introduction

A substantial proportion of the world’s land area is affected by soil salinity and alkalinity, particularly in arid and semi-arid regions, with an estimated global area of ~8.31–16.64 million square kilometers (Mkm^2^) (Munns & Tester 2008; Hassani et al. 2021; Negacz et al. 2022). For example, recent predictions suggested that the dryland areas of South America, southern Australia, and South Africa are at the risk of higher soil salinity caused by changing climate (Hassani et al. 2021). These soil constraints limit plant growth and pose serious risks to sustainable agricultural development and food security in the affected regions. In many fields within these regions, sodium carbonate (Na_2_CO_3_) and sodium bicarbonate (NaHCO_3_) accumulate in the soil profile, leading to the formation of alkaline soils with pH exceeding 8.5. High soil pH reduces water availability, limits nutrient acquisition, and promotes soil structural decline. These changes, referred as soil alkaline stress that is the major focus of this review, restrict root water uptake, slow plant growth and development, and ultimately reduce yield (Munir et al. 2021). In alkaline soils, while ion toxicity and the disruption of the osmotic balance that are the characteristics of neutral salt stress also occur, the high soil pH directly reduces the availability of essential macro- and micronutrients in the soil (e.g., nitrogen, phosphorus, iron, and manganese) (Neina, 2019; Rengel, 2015; Cao et al. 2022), which can lead to severe nutrient imbalances in plants. In addition, excess bicarbonate/carbonate (HCO_3_⁻/CO_3_^2^⁻) can interfere with ion transport, weaken membrane stability, and disturb intracellular pH regulation (Fang et al. 2021; Cao et al. 2022; Poschenrieder et al. 2018). Moreover, HCO_3_⁻ and CO_3_^2^⁻ can precipitate calcium (Ca^2^⁺) and magnesium (Mg^2^⁺) as insoluble carbonates, which further reduces nutrient availability and disrupts plant metabolism (Shrivastava & Kumar, 2015, Guo et al. 2015; Cao et al. 2022). Consequently, alkaline stress should not be regarded simply as neutral salt stress under higher pH. Rather, it represents a distinct alkaline stress that combines osmotic stress, Na⁺-associated ion toxicity, high-pH injury, impaired proton homeostasis, and more severe nutrient deficiency, and is therefore often more complex and damaging than neutral salt stress (Cao et al. 2022; Rao et al. 2023). Therefore, an in-depth investigation of plant responses to soil alkalinity is critical for enhancing crop resilience and productivity in alkaline salinity-affected regions.

Under the conditions of neutral salt stress, which is typically induced by neutral salts like sodium chloride (NaCl) and/or sodium sulfate (Na_2_SO_4_), plant growth inhibition occurs without significant alteration in soil pH, with stress being largely due to ion toxicity and disruption of the osmotic balance, resulting in reduced water uptake and disrupted plant nutrient balance (Fang et al. 2021; Munns & Tester 2008; Liang et al. 2024a). Therefore, plant responses to neutral salt stress mainly involve osmotic adjustment, Na⁺ exclusion or compartmentation, and maintenance of cytosolic sodium and potassium ion homeostasis (Na⁺/K⁺ homeostasis) (van Zelm et al. 2020; Liang et al. 2024a). Because the external pH in soils is not markedly elevated, neutral salt stress generally does not impose additional constraints on proton homeostasis and nutrient solubility that are the characteristics of alkaline soils (Li & Yang 2023).

In fact, neutral salt stress and alkaline stress often occur concurrently in natural soils, where neutral and alkaline salts coexist, as reflected by the existence of combined NaCl, Na_2_SO_4_, NaHCO_3_, and/or Na_2_CO_3_ (Fang et al. 2021; Li & Yang 2023; Rao et al. 2023). In this review, saline-alkali stress specifically denotes these combined conditions involving both neutral and alkaline salts, under which plants are simultaneously exposed to high salinity, elevated pH, variable anion composition, and reduced nutrient availability (Cao et al. 2022; Rao et al. 2023; Ma et al. 2026). The stress severity depends not only on total salt concentration, but also on the relative proportion of neutral and alkaline salts, and the resulting rhizosphere pH (Rao et al. 2023; Yang et al. 2024). Therefore, saline-alkali stress imposes overlapping but non-identical constraints compared with either neutral salt stress or alkaline stress alone. Plants exposed to saline-alkali stress must maintain osmotic adjustment and Na⁺/K⁺ homeostasis, while they must also sustain proton extrusion, rhizosphere acidification, organic acid secretion, and nutrient acquisition under high-pH conditions (Li & Yang 2023; Rao et al. 2023; Ma et al. 2026).

Among the plant species widely studied for dissecting mechanisms underlying plant salinity responses and explored as genetic resources in molecular breeding and biotechnology for improving salt tolerance, halophytes are particularly noteworthy. These species can complete their life cycles in saline-alkali habitats because they have evolved specialized physiological and molecular traits, including efficient ion homeostasis, pH regulation, osmotic adjustment, and antioxidative defenses. Based on their growth strategies under high salt conditions, halophytes are commonly classified as salt-accumulating euhalophytes, salt-secreting recretohalophytes, and salt-excluding pseudohalophytes (Mishra & Tanna 2017; Flowers & Colmer 2008; Rahman et al. 2021). Over the past two decades, extensive studies have elucidated a diverse range of responses of various plant species to NaCl-induced salt stress (Munns & Tester 2008; Deinlein et al. 2014; Liang et al. 2024b; Zhang et al. 2025a). Recent studies have also advanced our understanding of plant responses to alkaline stress across physiological, rhizosphere, molecular, and genetic levels (Rao et al. 2023; Cai et al. 2022; Ganapati et al. 2022; Fang et al. 2021; Ma et al. 2026). However, this evidence remains dispersed across different species, stress treatments, and experimental systems, making it necessary to synthesize current knowledge and distinguish alkaline stress associated responses from general salt stress mechanisms. In this review, we make an effort to summarize, integrate, and critically evaluate recent progresses in plant responses and tolerance to alkaline stress. First, we review the physiological foundations of alkaline stress tolerance in plants, focusing on pH regulation and the coordinated management of ion toxicity, reactive oxygen species (ROS) homeostasis, and osmotic adjustment. Second, we highlight how morphological traits, root system plasticity, and rhizosphere interactions reshape the root-soil microenvironment, complementing and supporting cellular regulation mechanisms that enable plants to cope with alkaline stress. Finally, we evaluate the molecular and genetic advances in plant alkaline stress tolerance, focusing on the coordination of transport, signaling, and transcriptional regulation of critical genes. We conclude by discussing how these mechanistic insights can guide breeding targets and accelerate the development of crop varieties that can be sustainably grown in alkaline soils.

## Physiological foundations of alkaline tolerance

In alkaline soils, plants face two linked constraints: Na⁺-associated ion toxicity and osmotic stress, and carbonate/bicarbonate-driven alkalinity that effectively reduces the external H⁺ supply and negatively impacts H⁺-coupled nutrient uptake (Chen et al. 2009; Li et al. 2010; Fang et al. 2021; Hasan et al. 2025). To maintain rhizosphere pH homeostasis under alkaline soil conditions, roots increase H⁺ extrusion through plasma membrane proton pumps and release organic acids to acidify the root zone, which partially counteracts soil alkalinity. To maintain cellular pH, the plasma membrane and tonoplast proton pumps stabilize the cytosolic pH and hence maintain the transmembrane proton motive force, thereby supporting cellular ion balance and metabolic stability (Shabala & Mackay 2011; Meng et al. 2018; Cao et al. 2022; Rahman et al. 2021). On the other hand, to cope with osmotic stress, ion toxicity, and excess ROS induced by alkaline conditions, plants activate numerous processes to maintain cellular ion balance, achieve osmotic adjustment, and scavenge excess ROS. These physiological responses are discussed in detail in this section.

### Proton pumps

**Fig. 1:**
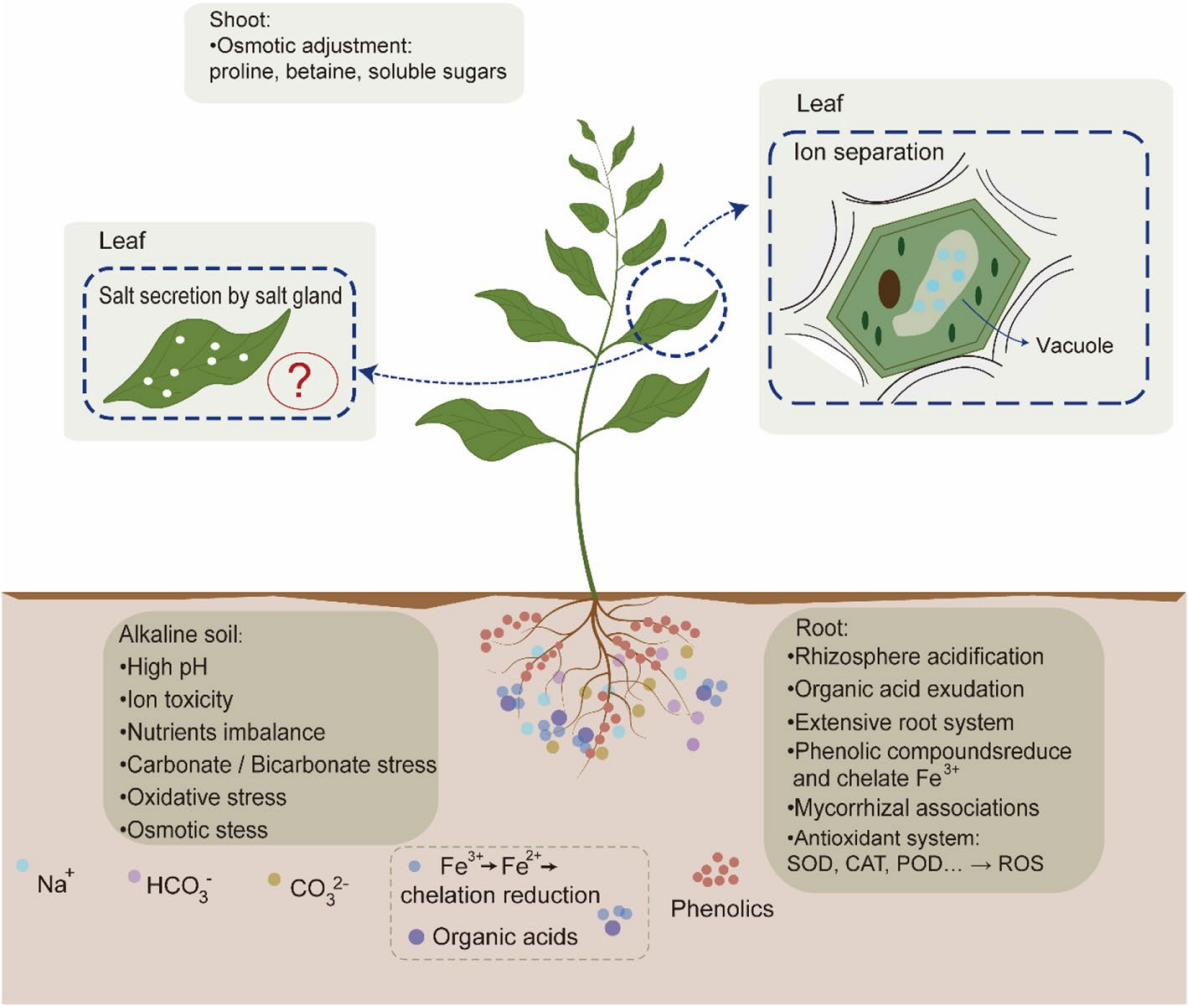
Schematic representation of physiological mechanisms underlying plant tolerance to alkaline stress. In alkaline soils, the accumulation of carbonate salts, such as Na_2_CO_3_ and NaHCO_3_, increases soil pH and elevates the concentrations of Na⁺, CO_3_^2^⁻, and HCO_3_⁻, while reducing the availability of nutrient elements, including Ca^2^⁺, Fe^2^⁺, Fe^3^⁺, and Mn^2^⁺. These soil conditions lead to osmotic stress, ion toxicity, and nutrient imbalance. In the rhizosphere, root organic acid exudation-induced acidification enhances the solubility and availability of micronutrients, such as Fe^2^⁺, Fe^3^⁺, and Mn^2^⁺. Additionally, citrate and malate acids chelate metal cations and partially neutralize CO_3_^2^⁻, HCO_3_⁻, thereby alleviating carbonate toxicity. Roots may also exude reducing or chelating substances (e.g., phenolics) into rhizosphere, thereby reducing Fe^3^⁺ to Fe^2^⁺ and chelating iron ions, further mobilizing sparingly soluble micronutrients in alkaline soils. Morphologically, some plant species develop extensive root systems to access deeper soil layers and form mycorrhizal associations, which significantly enhance nutrient absorption. In mesophyll cells, the accumulation of compatible solutes, such as proline, glycine betaine, soluble sugars, and certain sugar alcohols, helps maintain cellular water potential and protects proteins and membranes. Activities of antioxidant enzymes, including superoxide dismutase (SOD), ascorbate peroxidase (APX), catalase (CAT) and glutathione peroxidase (GPX), are enhanced, enabling coordinated scavenging of excess reactive oxygen species (ROS), thereby reducing lipid peroxidation and other oxidative damage. Increased ROS-scavenging action occurs in both shoots and roots in plants under alkaline stress. Whether salt glands or salt bladders directly contribute to alkaline tolerance remains unclear as indicated by a question mark in the scheme

To achieve alkaline tolerance, many halophytes have evolved a near-neutral cytosolic pH and a stable transmembrane proton motive force. Plants rely on a coordinated proton-pump system, including plasma membrane H⁺-ATPase (PM H⁺-ATPase), vacuolar H⁺-ATPase (V-H⁺-ATPase), and vacuolar H⁺-pyrophosphatase (V-H⁺-PPase) (Palmgren 2001; Falhof et al. 2016; Shabala & Mackay 2011; Meng et al. 2018; van Zelm et al. 2020). PM H⁺-ATPase uses ATP hydrolysis to pump H⁺ out of the cell. This activity builds an electrochemical gradient that drives H⁺-coupled uptake of nutrients (e.g., nitrate [NO_3_^−^] and phosphate [Pi]) and supports Na⁺/H⁺ exchange via Salt Overly Sensitive 1 (SOS1), which plays a critical role in pumping excess Na⁺ out of cells, thereby conferring a degree of salinity tolerance. A stronger membrane potential can reduce passive Na⁺ influx and support Na⁺ efflux. Continuous H⁺ extrusion also acidifies the rhizosphere, which partially offsets the weakening of the proton gradient at high external pH (Palmgren 2001; Shabala & Mackay 2011; van Zelm et al. 2020; Cao et al. 2022). These two functions place PM H⁺-ATPase at the frontline of alkaline tolerance by providing (i) the electrochemical driving force required for Na⁺/H⁺ exchange and (ii) the H⁺ efflux that acidifies the rhizosphere (Fig. 1).

Physiological and multi-omic studies suggest that higher activities of PM H⁺-ATPase, V-H⁺-ATPase, and V-H⁺-PPase, together with stronger Na⁺/H⁺ exchange, are associated with improved plant tolerance to alkaline stress (Meng et al. 2018; Rahman et al. 2021). In the alkaline-tolerant grass *Puccinellia tenuiflora*, Na_2_CO_3_ treatment increases the expression and activities of PM H⁺-ATPase, V-H⁺-ATPase, and V-H⁺-PPase. Na_2_CO_3_ treatment also induces the expression of genes encoding vacuolar Na⁺/H⁺ Exchangers (NHXs), which use the proton gradient to sequester Na⁺ and help regulate cellular pH. This coordinated upregulation is associated with increased Na⁺ sequestration and more stable cytosolic pH (Guo et al. 2010; Kobayashi et al. 2012; Zhang et al. 2013; Yu et al. 2013; Yin et al. 2019). Similar co-induction of proton pumps and Na⁺/H⁺ exchange has been observed in halophytes, such as *Suaeda salsa*, *Spartina alterniflora*, and *Zoysia matrella* (Fang et al. 2021; Rao et al. 2023)*.* Moreover, genetic manipulation of proton pumps and Na⁺ transport components in crops can improve their performance in alkaline soils (Baisakh et al. 2012; Chen et al. 2015c; Meng et al. 2018; Rahman et al. 2021; Han et al. 2005). Collectively, these findings support a model in which coordinated activation of PM H⁺ pumps drive rhizosphere acidification and, together with tonoplast NHXs, maintains cellular pH homeostasis and promotes Na⁺ compartmentation under alkaline stress.

### Organic acid secretion

Organic acid secretion is a well-documented strategy that supports alkaline tolerance in halophytes. Organic acids partly dissociate in the rhizosphere, release H⁺, and thereby help buffer the alkaline root zone. In *P. tenuiflora*, alkaline treatment increases citrate and malate levels in roots and the rhizosphere soil solution, triggering the rhizosphere pH shifts from strongly alkaline toward near-neutral levels (Guo et al. 2010). Integrated metabolomic and proteomic analyses have shown that under Na_2_CO_3_ stress, *P. tenuiflora* upregulates organic acid–related pathways, including the tricarboxylic acid (TCA) cycle, glyoxylate metabolism, and glycolysis. Upregulation of these pathways is accompanied by an increased abundance of multiple dehydrogenases and synthases involved in organic acid metabolism. Similar responses occur in several alkaline-tolerant species, including barley (*Hordeum vulgare*), alfalfa (*Medicago sativa*), *Leymus chinensis*, Yorkshire fog (*Holcus lanatus*), red clover (*Trifolium pratense*), and several forage grasses, with alkaline salt treatments inducing stronger organic acid accumulation and stronger rhizosphere buffering than neutral salt treatments (Yang et al. 2009; Li et al. 2010; Wang et al. 2024; Olea et al. 2025; Chen et al. 2024). Moreover, increases in organic acid contents are strongly correlated with root growth recovery and a reduced Na⁺/K⁺ ratio in barley and alfalfa plants (Yang et al. 2009; Li et al. 2010; Chen et al. 2024). Overall, metabolomic evidence suggests that plants actively redirect carbon metabolism toward organic acid synthesis and release under alkaline stress, rather than showing only passive accumulation of metabolic byproducts (Panda et al. 2021; Kumari et al. 2025; Chen et al. 2009; Yu et al. 2013; Yin et al. 2019; Wang et al. 2024) (Fig. 1).

After secretion, citrate and malate partially dissociate and release H⁺ into the rhizosphere. The released protons can neutralize alkaline stress, forming carbonic acid that decomposes into CO_2_ and H_2_O, thereby lowering rhizosphere pH. In addition, the carboxyl groups in organic acids can chelate cations, such as ferrous/ferric iron (Fe^2+^/Fe^3+^) and manganese (Mn^2+^), which can improve micronutrient availability at high soil pH. Furthermore, when the uptake of inorganic anions (e.g., NO_3_^−^, dihydrogen phosphate/hydrogen phosphate [H_2_PO_4_^−^/HPO_4_^2−^], and sulfate [SO_4_^2−^]) is limited, organic anions released from organic acids can help maintain both membrane charge balance and osmotic homeostasis (Gamarra Reinoso et al. 2024; Poschenrieder et al. 2018; Fang et al. 2021). In addition, increased organic acid secretion is often associated with enhanced root hair and lateral root formation. This results in a greater root absorptive surface and can support selective ion uptake and contribute to a lower Na⁺/K⁺ ratio (Guo et al. 2010; Fang et al. 2021; Rahman et al. 2021; Cao et al. 2022).

### Cellular ion balance

Under alkaline stress, cytosolic Na⁺ accumulation is a major threat to cell metabolic functions. Halophytes often use Na⁺ salts (including NaHCO_3_ and Na_2_CO_3_ in alkaline soils) as osmoticum, while still keeping cytosolic Na⁺ levels low and retaining K⁺ (Munns & Tester 2008; Flowers et al. 2015). Many halophytes rely on a combined strategy of vacuolar Na⁺ sequestration and cytosolic K⁺ retention. These plants accumulate Na⁺ salts in vacuoles to provide osmotic adjustment, while maintaining cytosolic K⁺ at near non-stress levels and cytosolic Na⁺ at low levels, thereby protecting metabolic function (Flowers et al. 2015; Meng et al. 2018). Vacuolar Na⁺/H⁺ exchangers cooperate with V-H⁺-ATPase and V-H⁺-PPase to move Na⁺ from the cytosol into the vacuole, where Na⁺ can contribute to osmotic balance without disrupting cytosolic processes. In *P. tenuiflora*, NaHCO_3_ and Na_2_CO_3_ treatments strongly induce vacuolar Na⁺/H⁺ exchange activity in parallel with vacuolar proton pumping. This coordinated response is associated with improved pH regulation and Na⁺ compartmentation under combined high-salt and high-pH conditions (Kobayashi et al. 2012). Similar responses have been observed in other saline-alkali tolerant grasses and halophytes, indicating that enhanced vacuolar Na⁺ sequestration is a common feature of alkaline tolerance (Guo et al. 2010; Meng et al. 2018; Rahman et al. 2021).

Under alkaline stress, membrane depolarization and ROS production can activate outward-rectifying K⁺ channels and trigger K⁺ efflux (Demidchik 2014; Demidchik et al. 2010; Mostofa et al. 2022). A characteristic feature of many halophytes is their ability to rapidly enhance PM H⁺-ATPase activity and H⁺ efflux (Chen et al. 2009; Bose et al. 2015). This response helps repolarize the membrane, suppress stress-induced K⁺ leakage, and maintain a strong driving force for high-affinity K⁺ uptake (Anschütz et al. 2014; Falhof et al. 2016). Studies under both salt and alkaline stresses have shown that halophytes maintain a much higher cytosolic K⁺/Na⁺ ratio than glycophytes (Zhang et al. 2013; Guo et al. 2015; Yang et al. 2009). This pattern suggests tighter coordination among proton pumps, K⁺ transport, and Na⁺/H⁺ exchange in halophytes (Chen et al. 2009; Bose et al. 2013; Meng et al. 2018). Although many mechanistic details come from NaCl studies (van Zelm et al. 2020), available evidence supports a similar ion-homeostasis framework under alkaline stress (Kobayashi et al. 2012; Guo et al. 2010) (Fig. 1).

### Osmotic adjustment

Osmotic adjustment is another important component of halophyte adaptation under alkaline stress. Alkaline stress reduces the external water potential and promotes the accumulation of Na⁺, together with accompanying anions (e.g., chloride [Cl⁻], SO_4_^2^⁻, and HCO_3_⁻/CO_3_^2^⁻). To maintain turgor pressure while avoiding ion toxicity, many halophytes use Na⁺ salts as a major vacuolar osmoticum and accumulate compatible solutes (e.g., proline, glycine betaine, soluble sugars, and sugar alcohols) in the cytosol and organelles. These solutes complement Na⁺ compartmentation and pH regulation (Flowers et al. 2015; Mishra & Tanna 2017; Meng et al. 2018). These solutes also stabilize membranes and proteins, preserve enzyme function, and support ROS control and redox balance (Bose et al. 2013; Panda et al. 2021; Kumari et al. 2025). Although many biosynthetic pathways were first characterized under NaCl stress, metabolomic studies in *P. tenuiflora*, wild soybean (*Glycine soja*), and other halophytes have shown that higher levels of proline, glycine betaine, and soluble sugars are also associated with improved growth under NaHCO_3_, Na_2_CO_3_, or alkaline treatments (Kumari et al. 2025; Panda et al. 2021; Chen et al. 2009; Yu et al. 2013; Yin et al. 2019). Therefore, osmotic adjustment likely supports alkaline tolerance but is not specific to alkaline stress response itself (Fig. 1).

### ROS homeostasis and antioxidant defenses

In alkaline environments, osmotic stress and ion toxicity often trigger excessive ROS production. If plants fail to remove ROS efficiently, ROS can cause lipid peroxidation, and protein and DNA oxidation (Fang et al. 2021; Pirasteh-Anosheh et al. 2023). Many glycophytes show ROS overaccumulation and oxidative damage under alkaline stresses (Yang et al. 2009; Guo et al. 2015), whereas many halophytes effectively control ROS levels under such stresses (Fang et al. 2021; Rao et al. 2023). Halophytes regulate ROS accumulation by maintaining ROS at levels sufficient for signaling, while deploying enzymatic and non-enzymatic antioxidant systems to prevent oxidative damage from excessive ROS accumulation. This control helps plants maintain ion balance, pH homeostasis, and osmotic adjustment (Bose et al. 2013; Rahman et al. 2021). Under alkaline stress, high apoplastic pH and micronutrient limitation (e.g., Fe deficiency) can further promote ROS formation, which makes ROS control especially important (Fig. 1).

Halophytes often show higher baseline activities of antioxidant enzymes and more rapidly increased levels of these enzymes than glycophytes under alkaline stress. Superoxide dismutase (SOD) catalyzes the first step of ROS detoxification by converting superoxide anion (O_2_·⁻) to hydrogen peroxide (H_2_O_2_). Catalase (CAT) and peroxidases (e.g., peroxidase [POD], ascorbate peroxidase [APX], glutathione peroxidase [GPX]) then detoxify H_2_O_2_ by converting it to water (and oxygen in the case of CAT), forming an efficient enzymatic scavenging cascade (Kohli et al. 2019; Pirasteh-Anosheh et al. 2023). In halophytes, such as *Atriplex* spp., *Suaeda* spp., and salt cress (*Eutrema salsugineum*), SOD, CAT, and POD activities under alkaline stress are often higher than those in glycophytes, whereas lipid peroxidation is lower. This contrast indicates that halophytes more effectively restrict ROS accumulation and limit oxidative damage (Bose et al. 2013; Rahman et al. 2021; Pirasteh-Anosheh et al. 2023).

Chloroplasts, mitochondria and peroxisomes are major intracellular ROS sources under alkaline stress, while plasma membrane–localized NADPH oxidases (respiratory burst oxidase homologs, RBOHs) are key enzymatic sources of ROS at the cell surface. Halophytes often maintain higher antioxidant activities in these organelles and tighter control of plasma membrane-associated ROS, which helps reduce local oxidative damage (Kohli et al. 2019; Bose et al. 2013).

In addition to enzymatic defenses, halophytes also rely on non-enzymatic antioxidants, including glutathione (GSH) and ascorbate (AsA), both directly act as cofactors of the AsA-GSH cycle, and phenolic compounds (e.g., flavonoids) (Rahman et al. 2021; Pirasteh-Anosheh et al. 2023). The AsA-GSH cycle uses enzymes, such as APX, monodehydroascorbate reductase (MDHAR), dehydroascorbate reductase (DHAR) and glutathione reductase (GR), to maintain high AsA/dehydroascorbate (DHA) and GSH/oxidized glutathione (GSSG) ratios (Hasanuzzaman et al. 2019; Foyer & Noctor 2011). This cycle is a major route for H_2_O_2_ detoxification in chloroplasts and the cytosol. Many studies have shown that halophytes retain higher GSH contents and higher GSH/GSSG ratios than glycophytes under alkaline stress. These redox buffers support ROS removal and provide reducing power for peroxidases (Rahman et al. 2021; Pirasteh-Anosheh et al. 2023; Srivastava et al. 2015). Flavonoids, including flavonols, flavanones, and anthocyanins, and other phenolics can accumulate in epidermal cells and vacuoles, where these compounds can scavenge ROS through hydrogen atom donation and metal ion chelation, thereby limiting oxidative damage under alkaline stress (Pirasteh-Anosheh et al. 2023). In many halophytes, alkaline stress induces higher total phenolics and flavonoids, and these increases often occur together with higher SOD, POD, and CAT activities. This pattern suggests that the above systems act together to enhance antioxidant capacity and restrain ROS accumulation (Bose et al. 2013; Rahman et al. 2021).

Overall, halophytes use an enzymatic scavenging system, centered on SOD, POD, and CAT, and non-enzymatic antioxidant system, e.g., GSH, AsA, and flavonoids, to regulate ROS under alkaline stress. This combined strategy supports short-term ROS signaling, while preventing long-term ROS overload, which would otherwise cause irreversible oxidative damage (Bose et al. 2013; Rahman et al. 2021; Mann et al. 2023).

Taken together, alkaline tolerance reflects a coordinated network in which proton-pump–driven pH homeostasis and organic acid–mediated rhizosphere buffering interface with Na⁺ compartmentation/K⁺ retention, ROS control, and osmotic adjustment to sustain cellular function in plants (Fig. 1). Addressing these factors either individually or in combination may enhance plant tolerance to alkaline stress.

## Morphology- and rhizosphere-associated regulation of alkaline tolerance

The physiological processes described above occur in specific cells, tissues and organs, and can consequently induce morphological changes in shoots and roots. For example, in many halophyte plant species, leaf and stem succulence is induced under saline-alkaline soil conditions by the accumulations of various secondary metabolites, thereby enhancing saline-alkaline tolerance (Flowers & Colmer 2008; Fang et al. 2021). Under alkaline stress, the ability of plants to maintain pH and ion homeostasis depends strongly on root-mediated soil exploration, rhizosphere modification, and interactions with associated microorganisms (Yang et al. 2024; Galindo-Castañeda et al. 2024; Araujo et al. 2025). Additionally, morphological traits, root architectural plasticity, organic acid exudation, microbial acidification, and mycorrhiza-assisted nutrient acquisition should be viewed as interconnected components of an integrated root–soil interface strategy rather than as isolated adaptive responses (Galindo-Castañeda et al. 2024; Boorboori & Lackóová, 2025). In this section, we discuss how plant morphology and rhizosphere interactions extend cellular alkaline-tolerance mechanisms to the whole-plant and root-soil-interface levels.

### Morphological plasticity and root-zone exploration

Halophytes growing in alkaline soils often show distinct morphological traits, including shoot succulence and, in some salt-secreting halophytes, salt glands or epidermal bladders that facilitate salt secretion or sequestration (Flowers & Colmer 2008; Shabala & Mackay 2011; Paz et al. 2012; Fang et al. 2021). Researchers widely recognize these traits as general adaptations to salinity and water deficit. However, we should not treat these traits as alkaline stress-specific unless experiments under such stress support that conclusion.

In succulent halophytes, such as *Suaeda* spp. and *Salicornia* spp., leaves and stems often contain large parenchyma cells with expanded vacuoles, reduced intercellular air spaces, and high tissue water content (Flowers et al. 2015). This tissue organization increases vacuolar capacity for Na⁺ storage and supports dilution and osmotic adjustment at the organ level. As a result, plants can maintain lower cytosolic ion levels and better cellular hydration in saline-alkali habitats (Meng et al. 2018; Fang et al. 2021; Paz et al. 2012). In salt-secreting halophytes, salt glands or bladders can excrete excess salts from leaves and young shoots. This feature is likely beneficial for maintaining leaf function in saline environments, but direct evidence under alkaline stress remains limited (Flowers and Colmer 2008; Shabala and Mackay 2011; Fang et al. 2021). Therefore, these traits are best described as habitat-associated phenotypes that may accompany alkaline stress tolerance, whereas mechanistic tolerance is more consistently explained by pH regulation and ion-transport homeostasis (Fig. 1).

Compared with shoot traits, root architectural plasticity is more directly connected to alkaline stress because root is the first organ exposed to high-pH soils and is responsible for rhizosphere acidification, nutrient uptake, and selective ion transport (Zeng et al. 2024; Galindo-Castañeda et al. 2024; Kumar et al. 2024). Changes in primary root growth, lateral root formation, and root hair development can alter the spatial domain of H⁺ extrusion and organic acid release, thereby influencing local pH buffering and nutrient mobilization (Zeng et al. 2024; Galindo-Castañeda et al. 2024). A more extensive or highly branched root system may increase the contact area for Fe, Pi, Mn, and other poorly available nutrients under alkaline conditions, whereas severe alkaline stress can suppress root elongation and reduce the capacity for ion and water uptake (Galindo-Castañeda et al. 2024; Kumar et al. 2024; Yang et al. 2024). Therefore, root morphological traits should be interpreted together with proton-pump activity, organic acid metabolism, and nutrient-acquisition pathways, rather than as independent tolerance traits (Zeng et al. 2024). In addition, extensive root systems and root-associated symbioses can enhance nutrient acquisition in alkaline soils, linking morphological plasticity with the rhizosphere and microbial mechanisms as discussed in the next subsection (Chen et al. 2016).

### Plant–microbe interactions as extensions of rhizosphere pH and nutrient regulation

At the root–soil interface, plant-associated microbes can reinforce several processes that plants use to tolerate alkaline stress. These include rhizosphere acidification, nutrient mobilization, osmotic adjustment, hormone regulation, and ROS control (Li et al. 2025; Nguyen et al. 2025; Boorboori & Lackóová, 2025). Rhizosphere bacteria associated with halophytes often tolerate both salinity and alkalinity, and these bacteria can support plant growth and soil structure and function in saline-alkali ecosystems (Egamberdieva et al. 2019; Rahman et al. 2021; Msimbira & Smith 2020; Kabir et al. 2026) Previous investigations indicated that plant growth-promoting rhizobacteria (PGPRs) from saline-alkali soils can secrete organic acids and extracellular polysaccharides (EPSs). The activities of PGPRs lower rhizosphere pH, promote calcium carbonate dissolution, and improve the availability of nutrients, such as Fe and Pi in alkaline soils (Han et al. 2025; Li et al. 2024). PGPRs and EPSs can also promote stable soil aggregates and increase soil water-holding capacity, and these changes create a more favorable physical environment for root growth. In addition, many PGPRs also show traits, such as Pi solubilization, improved Fe and zinc (Zn) availability, phytohormone production, and 1-aminocyclopropane-1-carboxylate (ACC) deaminase activity. These traits can reshape root architecture and reduce stress-related ethylene accumulation, thereby supporting osmotic balance and redox balance under alkaline stress (Egamberdieva et al. 2019; Gao et al. 2022; Cui et al. 2025; Kabir et al. 2026). High-throughput sequencing studies in halophytes, such as *S. europaea*, *S. salsa*, and *Atriplex* spp., show that rhizosphere microbial communities differ from those in adjacent bulk soils, indicating plant-associated microbial assembly in saline-alkali soils. (Yuan et al. 2016; Yamamoto et al. 2018; Alfradique Monteiro et al. 2022). These microbial communities are often enriched in functions related to EPS synthesis, osmotic adjustment, and antioxidant functions. This functional enrichment is consistent with a long-term association between halophytes and their associated microbes in saline-alkali soils (Rahman et al. 2021).

Beyond PGPRs, mycorrhizal associations (especially with arbuscular mycorrhizal fungi, AMFs) can contribute to plant performance on alkaline soils by expanding the effective soil exploration volume and enhancing the acquisition of nutrients with low availability at high pH. Importantly, predictable relationships between below-ground traits and nutrient acquisition emerge only when both root morphology and mycorrhizal fungi are considered together (Chen et al. 2016). Under alkaline stress, AMF inoculation alleviated growth inhibition and improved osmotic regulation and ion balance in *L. chinensis* seedlings (Wang et al. 2022). Similar beneficial effects of AMF inoculation have also been reported under saline-alkali conditions in poplar (*Populus simonii* × *P. nigra*) (Dong et al. 2023).

Overall, rhizosphere microbes act as functional extensions of plant alkaline-tolerance mechanisms at the root–soil interface (Li et al. 2025; Nguyen et al. 2025). Microbial organic acids can act in parallel with plant-derived citrate, malate, and oxalate to lower rhizosphere pH and mobilize Fe, Pi, and other poorly available nutrients (Nguyen et al. 2025). Microbial EPSs improve soil aggregation and water retention, thereby indirectly supporting root water uptake and osmotic adjustment (Li et al. 2025; Boorboori & Lackóová, 2025). In addition, microbial ACC deaminase can contribute to reshaping root architecture and reducing stress-induced ethylene accumulation in plants, linking microbial activity to phytohormone signaling, lateral root development, and nutrient foraging (Li et al. 2025; Nguyen et al. 2025). AMFs further extend the effective absorptive surface of roots and can reinforce ion balance and osmotic regulation under alkaline stress (Boorboori & Lackóová, 2025). Thus, plant–microbe interactions are also mechanistically connected to the well-known processes associated with alkaline tolerance, including rhizosphere acidification, nutrient acquisition, osmotic adjustment, ion homeostasis, and ROS control, to improve plant performance under alkaline stress, rather than representing an independent tolerance layer.

## Molecular basis of plant alkaline tolerance

The physiological and rhizosphere responses described above are initiated by early perception systems that detect changes in the extracellular environment. Under alkaline stress, the most immediate extracellular changes include elevated rhizosphere and apoplastic pH, reduced proton availability, HCO_3_⁻/CO_3_^2^⁻ accumulation, altered plasma-membrane proton gradients, and changes in ion fluxes that can trigger Ca^2^⁺ and ROS signaling (Liu et al. 2022; Xu & Yu 2023; Li & Yang 2023; Vélez-Bermúdez et al. 2024). However, compared with downstream responses, such as PM H⁺-ATPase activation, Na⁺/H⁺ exchange, organic acid secretion, antioxidant defense, and transcriptional regulation, the initial perception of alkaline soil conditions by plants remains less well defined (Li & Yang 2023; Rao et al. 2023; Yang et al. 2024; Zeng et al. 2024). Therefore, early extracellular pH sensing and signal initiation represent a critical link between alkaline soil and the downstream pathways in plants as discussed below.

### Early perception of extracellular alkalinity and associated signals

Initial perception of alkaline stress should be distinguished from downstream alkaline stress-responsive physiological and molecular responses. Although plants clearly respond to NaHCO_3_/Na_2_CO_3_ treatments by activating H⁺ extrusion, organic acid metabolism, Na⁺/H⁺ exchange, ROS regulation, and stress-responsive transcriptional networks, these downstream responses do not necessarily identify the primary sensors of alkaline stress (Fang et al. 2021; Cao et al. 2022; Li & Yang 2023; Rao et al. 2023). At present, dedicated HCO_3_⁻/CO_3_^2^⁻ receptors have not been identified in plants. Thus, currently available lines of evidence more strongly support extracellular pH sensing, proton-gradient disturbance, membrane-potential changes, and secondary Ca^2^⁺/ROS signals as plausible early events rather than they support a defined HCO_3_⁻/CO_3_^2^⁻-specific receptor pathway (Xu & Yu 2023; Vélez-Bermúdez et al. 2024; Ma et al. 2026).

Previous investigations suggested that early alkalinity perception and signal initiation may involve extracellular pH sensing together with changes in proton gradients, membrane potential, and Ca^2^⁺/ROS signaling (Xu & Yu 2023; Vélez-Bermúdez & Schmidt 2023; Ma et al. 2026). Peptide receptor complexes at the cell surface can sense extracellular pH, providing an example of apoplastic pH perception. In this system, two peptide receptor complexes, Root Meristem Growth Factor 1-Root Meristem Growth Factor Receptor (RGF1-RGFR) and Plant Elicitor Peptide 1-Plant Elicitor Peptide Receptor (Pep1-PEPR), perceive extracellular pH through pH-dependent peptide receptor interactions and link apoplastic pH status to root meristem growth and immune signaling (Liu et al. 2022). This finding is highly relevant to alkaline-stress biology because alkaline soils impose sustained high-pH conditions on rhizosphere and root apoplast (Li & Yang 2023; Vélez-Bermúdez et al. 2024). Nevertheless, extracellular pH sensing has so far been studied mainly in developmental and immune contexts, not under alkaline stress. Therefore, whether RGF1-RGFR, Pep1-PEPR, or related cell-surface receptor systems participate directly in high pH perception under alkaline stress remains an open question (Liu et al. 2022; Xu & Yu 2023; Vélez-Bermúdez et al. 2024). Future studies should first test whether candidate cell surface receptors perceive extracellular pH under NaHCO_3_/Na_2_CO_3_-based alkaline treatments. This could be done using receptor mutants, apoplastic pH imaging in specific root cell types, and assays of pH dependent peptide receptor interactions.

### Ion-transport modules are reinforced under alkaline stress

**Fig. 2:**
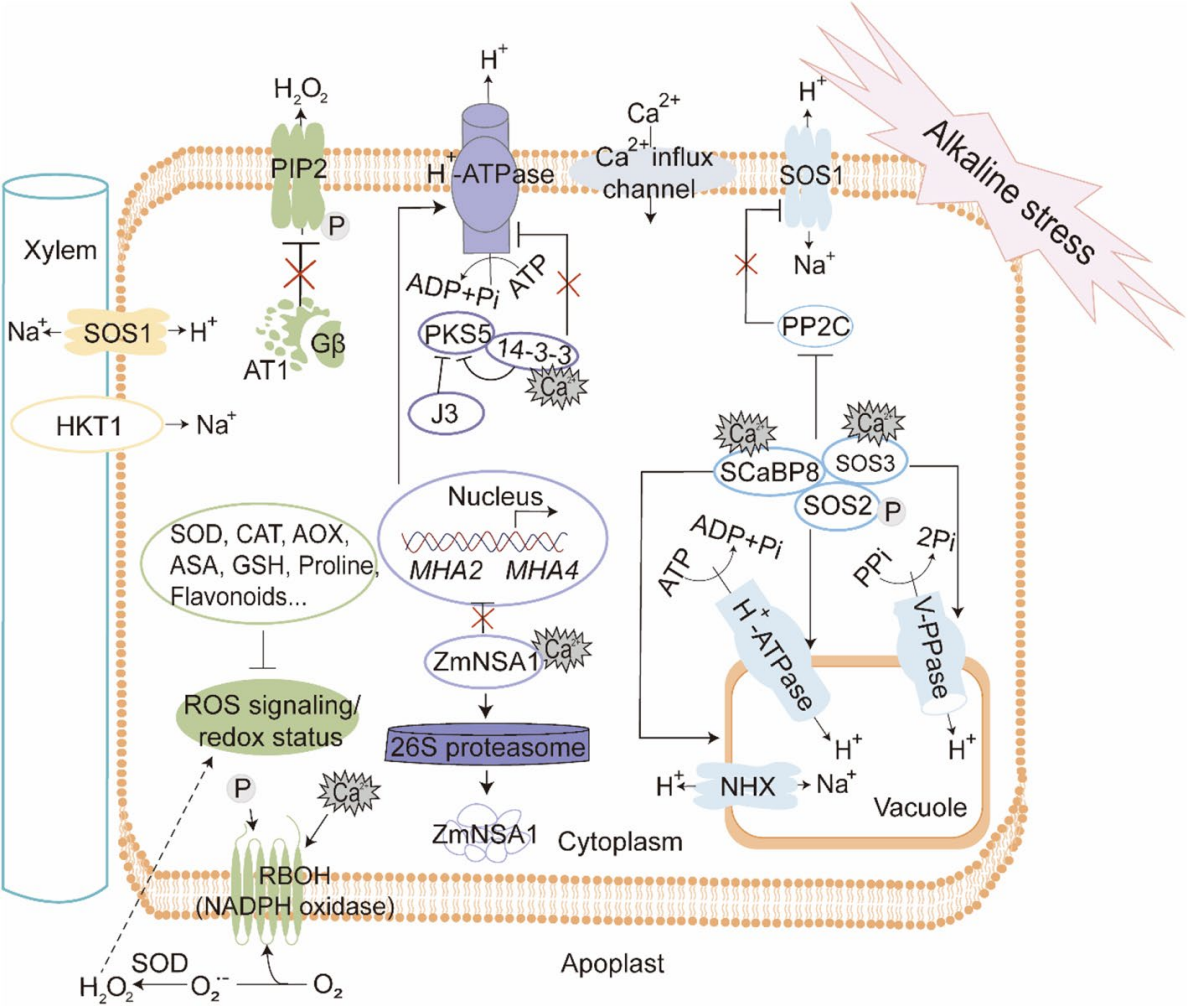
Genetic factors and biochemical pathways influencing tolerance to alkaline stress in plants. Alkaline stress induces a cytosolic Ca^2^⁺ influx. At the xylem–parenchyma interface, High-Affinity K⁺ Transporter 1 (HKT1) transporters retrieve Na⁺ to restrict its long-distance transport to the shoots, while the plasma-membrane Na⁺/H⁺ antiporter SOS1 mediates Na⁺ efflux. Ca^2^⁺-dependent regulators, such as the EF-hand protein Na⁺ Content Under Saline-Alkaline Condition 1 (ZmNSA1) in *Zea mays*, further modulate plasma membrane H⁺-ATPase (PM H⁺-ATPase) phosphorylation and activity, influencing Na⁺ exclusion from roots and Na⁺ accumulation in shoots under NaHCO_3_ stress. DnaJ Homolog 3 (J3) also interacts with Salt Overly Sensitive 2 (SOS2)-like Protein Kinase 5 (PKS5) under alkaline stress, inhibiting its kinase activity and relieving suppression of PM H⁺-ATPase. Enhanced H⁺-ATPase activity increases the proton motive force, supports SOS1 function, and promotes H⁺ efflux and rhizosphere acidification under alkaline conditions. The SOS3 and SOS3-like Ca^2^⁺-binding Protein 8 (SCaBP8) perceive Ca^2^⁺ signals, relieve the C-terminal autoinhibition of the protein kinase SOS2, and recruit SOS2 to the plasma membrane. The SCaBP8/SOS3–SOS2 kinase complex phosphorylates and activates SOS1, thereby enhancing Na⁺ efflux. Plasma membrane-localized SCaBP8 further inhibits the activity of Type 2 C Protein Phosphatases (PP2C) under alkaline stress, releasing SOS1 from negative regulation. Ca^2^⁺ signals also interact with 14–3-3 proteins, which further interact with and suppress PKS5, thereby activating PM H⁺-ATPases. In the vacuole, vacuolar H⁺-ATPase (V-H⁺-ATPase) and vacuolar H⁺-pyrophosphatase (V-H⁺-PPase) generate the proton gradient that drives NHX-type Na⁺/H⁺ antiporters to sequester excess Na⁺ into vacuoles. Respiratory burst oxidase homologs (RBOHs) mediate ROS production, which activates multiple antioxidant defense pathways. The atypical G protein γ subunit AT1/GS3 shapes the cellular redox environment by modulating the phosphorylation of Plasma Membrane Intrinsic Protein 2 (PIP2) aquaporins and the efflux of hydrogen peroxide (H_2_O_2_) into the apoplast. Together, these processes coordinately maintain ion and pH homeostasis and redox balance under alkaline stress

Following early perception of extracellular alkalinity, plants activate ion-transport modules together with alkaline-responsive regulatory pathways to achieve alkaline tolerance. Classical components include the Ca^2^⁺-dependent SOS pathway, vacuolar NHXs, xylem Na⁺ transport systems, such as the High-Affinity K⁺ Transporter (HKT) family members involved in xylem Na⁺ retrieval, and proton pumps that energize H⁺-coupled transport. However, most mechanistic insights into these modules come from studies under NaCl stress (Ji et al. 2013; Yang & Guo 2017; van Zelm et al. 2020). Under alkaline stress, alkaline-tolerant plant species often activate these classical transport and proton pump mechanisms more strongly and in a more coordinated manner, reflecting the need to maintain ion homeostasis, while preserving intracellular pH under high external pH (Fig. 2).

In the extremophile grass *P. tenuiflora*, integrated transcriptome and functional analyses under Na_2_CO_3_ stress show that PM SOS1-type Na⁺/H⁺ antiporters and NHXs are strongly induced together with proton pumps. This co-induction is associated with enhanced root Na⁺ efflux, increased vacuolar Na⁺ sequestration, and more stable cytosolic pH. In addition, these proteins often show higher activities than those in glycophytes under the same Na_2_CO_3_ treatment (Kobayashi et al. 2012; Zhang et al. 2013; Zhao et al. 2016). These results suggest that alkaline-tolerant plant species use the same core transport systems as NaCl-tolerant plants but more strongly upregulate and activate them under alkaline stress.

Beyond the classical transporters described above, recently identified regulatory modules modules further adjust proton pumping and Na⁺ homeostasis under alkaline stress. In *Arabidopsis thaliana*, SOS2-like Protein Kinase 5 (PKS5) associates with PM H⁺-ATPases to modulate H⁺ efflux, thereby supporting root acclimation to elevated pH (Yang et al. 2019). In addition, Cytosolic ABA Receptor Kinase 1 and 3 (CARK1 and CARK3) phosphorylate Arabidopsis Plasma Membrane H⁺-ATPase 2 (AHA2) at Thr469. This phosphorylation enhances AHA2 activity, H⁺ efflux, and alkaline-stress tolerance, further supporting the importance of phosphorylation-dependent PM H⁺-ATPase regulation under alkaline stress (Li et al. 2026). In tomato (*Solanum lycopersicum*), a 14–3-3 protein (TFT4) integrates H⁺ efflux with the PKS5–DnaJ Homolog 3 (J3) pathway during alkaline-stress response, providing an example of proton-pump regulation under high-pH conditions (Xu et al. 2013). In wheat (*Triticum aestivum*), Ca^2^⁺-dependent protein C-Terminal Centrin-Like Domain-Containing Protein 1 (TaCCD1) and Small Auxin Up RNA 215 (TaSAUR215) enhance PM H⁺-ATPase activity by limiting dephosphorylation by D-clade Type 2 C Protein Phosphatases (PP2C.D1/8), strengthening rhizosphere acidification and alkaline tolerance (Cui et al. 2023) (Fig. 2** and **Table 1). These studies support the importance of proton pump regulation in alkaline tolerance, although whether the same regulatory modules operate under neutral NaCl/Na_2_SO_4_ stress remains unclear.

**Table 1:** Representative genes and their roles in alkaline resistance from different plant species (2010–2025)

Plant species	Gene name	Alkali treatment conditions	Proposed mechanisms	References
*Arabidopsis thaliana*	*CBL10*	3.6 mM NaHCO_3_ (pH 7.7) and 7.4 mM NaHCO_3_ (pH 8.1)	Inhibition of PM H^+^-ATPase activity (AHA4/AHA11) modulates H^+^ efflux and pH homeostasis	(Xie et al. 2022)
*A. thaliana*	*EBS1*	3 mM NaHCO_3_	Positive regulation of bicarbonate tolerance via brassinosteroid signaling	(Chen et al. 2021)
*A. thaliana*	*GRF6* (*14–3-3*)	Alkaline pH treatment (pH 8.2; pH adjusted)	GRF6 (14–3-3) acetylation modulates its interaction with AHA2; deacetylation promotes H^+^ extrusion	(Guo et al. 2022)
*A. thaliana*	*MNS1/2/3* (*MNSs*)	Alkaline pH treatment (pH 8.2; pH adjusted)	N-glycan processing maintains auxin homeostasis and supports root growth	(Xia et al. 2022)
*A. thaliana*	*PIN2*	KOH-adjusted alkaline pH treatment (pH 8.0; adjusted daily with 0.1 mM KOH)	*PIN2* integrates basipetal auxin transport with H^+^ efflux in root tips via the PKS5–J3 module, promoting root growth	(Xu et al. 2012)
*A. thaliana*	*SCaBP3*	0, 1.5, 2, 4, and 6 mM NaHCO_3_ (pH 5.8, 6.55, 6.66, 6.80 and 6.93)	Increased PM H^+^-ATPase activity (AHA2 Thr947 phosphorylation) and H^+^ extrusion	(Yang et al. 2019)
*Glycine max*	*GmHXK15*	100 mM NaHCO_3_	Osmotic adjustment (carbon/energy metabolism): NaHCO_3_-induced HXK activity; improved root elongation and root fresh weight	(Jiao et al. 2023)
*G. max*	*GmMAX2a*	0.5–0.7 mM NaHCO_3_ (*A. thaliana*) and 50 mM NaHCO_3_ (*G. max*)	Strigolactone signaling improves growth and stress tolerance through hormone crosstalk and redox regulation	(Nisa et al. 2023)
*G. max*	*GmMYB14*	0.5–4.5 mM mixed alkali salts (Na_2_CO_3_:NaHCO_3_ = 1:1 pH 7.27 to 9.62)	Activates phenylpropanoid metabolism to mitigate ROS burst and improve Fe bioavailability, alleviating chlorosis	(Zhang et al. 2025a, b)
*G. max*	*GmNHX6*	8 and 90 mM NaHCO_3_	Maintains Na^+^/K^+^ homeostasis via Na^+^/H^+^ exchange	(Jin et al. 2022)
*Glycine soja*	*GsACA1*	50 mM NaHCO_3_ (pH 8.5; *A. thaliana*)	Maintained Ca^2+^ transport, homeostasis and reduced stress damage	(Sun et al. 2016a)
*G. soja*	*GsBOR2*	50 mM NaHCO_3_ (*A. thaliana*)	Improved boron transport and nutrient/ion balance	(Duan et al. 2018b)
*G. soja*	*GsbZIP43*	NaHCO_3_ (*A. thaliana*, soybean hairy roots)	Enhanced antioxidant enzyme activities (SOD, POD, CAT) and reduced MDA accumulation; improved Na^+^/K^+^ ratio	(Jia et al. 2024)
*G. soja*	*GsCHX19.3*	50 mM NaHCO_3_ (Arabidopsis)	Improved cellular pH regulation and cation balance via cation/H^+^ exchange	(Jia et al. 2017)
*G. soja*	*GsCML27*	50 mM NaHCO_3_ (pH 8.5; *A. thaliana*)	Maintains Na^+^/K^+^ ion balance and osmotic-stress tolerance	(Chen et al. 2015b)
*G. soja*	*GsCPI14*	50 mM NaHCO_3_ (pH 8.5; Arabidopsis)	Reduced oxidative damage and improved growth via GsCPI14–GsCBRLK module	(Sun et al. 2014)
*G. soja*	*GsEARLI17*	8 mM NaHCO_3_ (germination rate) and 150 mM NaHCO_3_ (soil irrigation; *A. thaliana*)	Osmotic adjustment; secreted cell wall protein affecting cuticle synthesis and leaf development	(Liu et al. 2015)
*G. soja*	*GsERF71*	50 mM NaHCO_3_ (pH 8.5; *A. thaliana*)	Elevated PM H^+^-ATPase activity and H^+^ extrusion	(Yu et al. 2017)
*G. soja*	*Gshdz4*	50 mM NaHCO_3_ (*A. thaliana*)	Reduced MDA contents but higher POD active	(Cao et al. 2016)
*G. soja*	*GsJAZ2*	150 mM NaHCO_3_ (*G. soja*)	Higher compatible solutes (e.g., proline/soluble sugars) and improved growth	(Zhao et al. 2020)
*G. soja*	*GsMIOX1a*	50 mM NaHCO_3_ (*A. thaliana*)	Lower MDA but higher POD activity; reduced oxidative damage	(Chen et al. 2015a)
*G. soja*	*GsMSRB5a*, *GsCBRLK*	50 mM NaHCO_3_ (pH 8.5; *A. thaliana*)	Reduced H_2_O_2_/MDA and higher antioxidant enzyme activities	(Sun et al. 2016b)
*G. soja*	*GsNAC019*	7, 9 or 50 mM NaHCO_3_ (pH 8.5) and 150 mM NaHCO_3_ (*A. thaliana*)	Enhanced PM H^+^-ATPase (AHA2) expression/activity and root H^+^ extrusion; induced expression of stress-related genes	(Cao et al. 2017)
*G. soja*	*GsSLAH3*	50 mM NaHCO_3_ (*A. thaliana*)	Adjusted anion flux (e.g., NO_3_^−^/Cl^−^) to stabilize membrane potential and ion balance	(Duan et al. 2018a)
*G. soja*	*GsSnRK1.1*,* GsSnRK1.2*	30 mM NaHCO_3_	Osmotic adjustment (metabolic regulation): NaHCO_3_-responsive SnRK1 activation/expression associated with stress adaptation	(Chen et al. 2021)
*G. soja*	*GsTIFY10*	50 mM NaHCO_3_ (*A. thaliana*)	Lower ROS/MDA and higher osmolyte accumulation, improving seedling performance	(Zhu et al. 2011)
*G. soja*	*GsWRKY15*	150 mM NaHCO_3_ (*Medicago sativa*)	Higher chlorophyll but lower MDA; induced NADP-ME and H^+^-PPase	(Zhu et al. 2017)
*Leymus chinensis*	*LcTprxII*	75 mM Na_2_CO_3_ (*Z. mays*)	Lower H_2_O_2_ and MDA but higher SOD/CAT/POD activities, maintaining chlorophyll and growth	(Michael et al. 2025)
*Malus domestica*	*MdSINA2*, *MdNAC104*	NaHCO_3_:Na_2_CO_3_ = 1:1 (pH 9.0)	Ubiquitination-mediated transcriptional regulation modulates GABA metabolism and malate transport to support pH balance	(Li et al. 2024)
*Oryza sativa*	*osa-MIR396c*	75 mM NaHCO_3_	miR396c targets GRF transcription factors; overexpression decreases salt and alkali stress tolerance	(Gao et al. 2010)
*O. sativa*	*OsGS3*	75 mM mixed alkali salts (NaHCO_3_:Na_2_CO_3_ = 5:1) and 62.5 mM NaHCO_3_ and 12.5 mM Na_2_CO_3_; pH 9.2 to 9.4	AT1/GS3 restricts PIP2-mediated H_2_O_2_ efflux; loss-of-function enhances H_2_O_2_ export and reduces intracellular ROS	(Zhang et al. 2023)
*O. sativa*	*OsIRO2, OsIRT1, OsNAS1, OsNAS2, OsYSL2, OsYSL15*	10 mM Na_2_CO_3_	Acquisition of iron rhizospheric acidification and phytosiderophore exudation	(Li et al. 2016)
*O. sativa*	*OsJRL, OsPEX11, OsNAC9, OsAKT1*	15 mM Na_2_CO_3_	Modulates Na^+^/K^+^ transport and reduces ROS accumulation	(Liu et al. 2019)
*O. sativa*	*OsLOX10*	25 mM Na_2_CO_3_ (pH 10.0)	Enhanced oxylipin (9-LOX) signaling improves ROS scavenging and stress tolerance	(Wang et al. 2023)
*O. sativa*	*OsPPa6*	50 mM mixed alkali (NaHCO_3_/Na_2_CO_3_ = 9:1; pH 9.17)	Lower Na^+^/K^+^ ratio; reduced MDA; higher soluble sugars and proline	(Wang et al. 2019)
*O. sativa*	*PhyB, AMT1*	80 mM NaHCO_3_ (*A. thaliana*)	Regulates NH_4_^+^ transport	(Jung et al. 2023)
*Solanum lycopersicum*	TFT4	Alkaline stress (pH 7.5, 7.8, 8.0, or 8.2)	Regulates PM H^+^-ATPase activity through 14–3-3 interaction, enhancing H^+^ efflux and supporting root growth	(Xu et al. 2013)
*Sorghum bicolor*	*SbAT1*	75 mM mixed alkali salts (NaHCO_3_:Na_2_CO_3_ = 5:1) and 62.5 mM NaHCO_3_ and 12.5 mM Na_2_CO_3_; pH 9.2 to 9.4	AT1/GS3 restricts PIP2-mediated H_2_O_2_ efflux; loss-of-function enhances H_2_O_2_ export and reduces intracellular ROS	(Zhang et al. 2023)
*Suaeda corniculata*	*ScVHA-B*, *ScVHA-C and ScVHA-H*	100 mmol L^–1^ NaHCO_3_	Supports vacuolar H^+^-ATPase activity	(Wang et al. 2016)
*S. corniculata*	*ScVP*	10.0 mM NaHCO_3_ and 90 mM NaCl (*A. thaliana*)	Enhanced vacuolar acidification (higher H^+^-PPase activity), supporting ion compartmentation	(Liu et al. 2011)
*Triticum aestivum*	*TaPP2C.D1/8*, *TaHA2*, *TaCCD1*,* TaSAUR215*	60 and 100 mM mixed alkali (NaHCO_3_:Na_2_CO_3_ = 9:1)	Activates H^+^-ATPase (TaHA2) via Ca^2+^-Maintained HA2 phosphorylation and higher PM H^+^-ATPase activity, enhancing H^+^ extrusion under mixed alkali stress	(Cui et al. 2023)
*Vitis vinifera*	*PEPC/PEPCK, ALMT*	75 mM NaHCO_3_ (pH 8.7)	Candidate genes associated with H^+^ pumping, organic acid transport, and auxin-related processes implicated in tolerance	(Xiang et al. 2019)
*V. vinifera*	*VvERF1B*, *VvMYC2*,* VvPMA10*	100 mM NaHCO_3_ for seedlings, and 2.5 and 100 mM NaHCO_3_ for Calli	High PM H^+^-ATPase activity to promote H^+^ efflux and oxalate secretion	(Xiang et al. 2025)
*Zea mays*	*ZmNSA1*	100 mM NaCl and 100 mM NaHCO_3_	Promotes root H^+^ efflux and SOS1-mediated Na^+^ efflux via the 26S proteasome pathway	(Cao et al. 2020)

*CBL10* Calcineurin B-like protein 10, *EBS1* S-ribonuclease binding protein EBS1, *GRF6 (14–3-3)* General regulatory factor 6 (14–3-3 protein), *MNS1/2/3 (MNSs)* α-mannosidase 1/2/3, *PIN2* PIN-FORMED 2, *SCaBP3* SOS3-like calcium-binding protein 3, *GmHXK15* Hexokinase 15, *GmMAX2a* More axillary growth 2a, *GmMYB14* MYB transcription factor 14, *GmNHX6* Na^+^/H^+^ exchanger 6, *GsACA1* Autoinhibited Ca^2+^-ATPase 1, *GsBOR2* Boron transporter 2, *GsCHX19.3* Cation/H^+^ exchanger 19.3, *GsCML27* Calcium-binding EF-hand protein 27, *GsCPI14* Cysteine proteinase inhibitor 14, *GsEARLI17* Early Arabidopsis aluminium-induced 17 (secreted cell wall protein), *GsERF71* Ethylene response factor 71, *Gshdz4* Homeodomain-leucine zipper (HD-Zip) I transcription factor, *GsJAZ2* Jasmonate ZIM-domain protein 2, *GsMIOX1a* myo-inositol oxygenase 1a, *GsMSRB5a* Methionine sulfoxide reductase B5a, *GsCBRLK* Calcium/calmodulin-binding receptor-like kinase, *GsNAC019* NAC transcription factor 019, *GsSLAH3* Slow-type anion channel homolog 3, *GsSnRK1.1* SNF1-related protein kinase 1.1, *GsSnRK1.2* SNF1-related protein kinase 1.2, *GsTIFY10* TIFY family protein 10, *GsWRKY15* WRKY transcription factor 15, *LcTprxII* Type II peroxiredoxin, *MdSINA2* Seven in absentia E3 ubiquitin ligase 2, *MdNAC104* NAC transcription factor 104, *osa-MIR396c* Oryza sativa microRNA396c, *OsGS3* Grain size 3, *OsIRO2* iron-related transcription factor 2, *OsIRT1* Iron-regulated transporter 1, *OsNAS1*, Nicotianamine synthase 1, *OsNAS2* Nicotianamine synthase 2, *OsYSL2* Yellow stripe-like transporter 2, *OsYSL15* Yellow stripe-like transporter 15, *OsJRL* Jacalin-related lectin, *OsPEX11* Peroxisome biogenesis factor 11 (peroxin 11), *OsNAC9* NAC transcription factor 9, *OsAKT1* Shaker-like K^+^ channel 1, *OsLOX10* Lipoxygenase 10, *OsPPa6* Soluble inorganic pyrophosphatase 6, *PhyB* Phytochrome B, *AMT1* Ammonium transporter 1, *TFT4* 14–3-3 protein TFT4, *SbAT1* Alkaline tolerance 1, *ScVHA-B* V-H^+^-ATPase subunit B, *ScVHA-C* V-H^+^-ATPase subunit C, *ScVHA-H* V-H^+^-ATPase subunit H, *ScVP* Vacuolar H^+^-pyrophosphatase, *TaPP2C.D1/8* Protein phosphatase 2 C (clade D1/8), *TaHA2* Plasma membrane H^+^-ATPase 2, *TaCCD1* C-terminal centrin-like domain containing protein 1, *TaSAUR215* Small auxin upregulated RNA 215, *PEPC/PEPCK* Phosphoenolpyruvate carboxylase/phosphoenolpyruvate carboxykinase, *ALMT* Aluminum-activated malate transporter, *VvERF1B* Ethylene response factor 1B, *VvMYC2* Basic helix-loop-helix transcription factor MYC2, *VvPMA10* Plasma membrane H^+^-ATPase 10, *ZmNSA1* Na^+^ content under saline-alkaline condition 1 (EF-hand Ca^2+^-binding protein)

Additional transport-related and Ca^2^⁺-linked components identified in *G. soja* also fit within this broader ion-transport framework (Fang et al. 2021; Cao et al. 2022). For example, *Ca*^*2*^*⁺*-*Binding EF-Hand Protein 27* (*GsCML27*) enhances tolerance to alkaline stress but reduces tolerance to NaCl and osmotic stress when ectopically expressed in Arabidopsis, suggesting that the effects of Ca^2^⁺-binding proteins depend on the type of abiotic stress (Chen et al. 2015b; Fang et al. 2021). *Slow Anion Channel-Associated Homolog 3* (*GsSLAH3*) positively modulates tolerance to bicarbonate-based alkaline stress, indicating that anion transport can contribute to plant responses under alkaline conditions (Duan et al. 2018a; Cao et al. 2022). Similarly, *Boron Efflux Transporter 2* (*GsBOR2*) improves bicarbonate tolerance when heterologously expressed in Arabidopsis (Duan et al. 2018b). Together, these examples indicate that Ca^2^⁺-linked regulation and anion transport support pH regulation and anion balance under alkaline stress (Fang et al. 2021; Cao et al. 2022) (Table 1).

### Genetic diversity and crop-specific adaptive traits for alkaline-soil breeding

Genetic diversity in crops is shaped by natural variation, domestication, and crop improvement. Genetic resources from major crops, wild relatives, landraces, and breeding populations provide an important but still underused route for improving alkaline-soil adaptation. Unlike single-gene overexpression studies, allelic variation identified in tolerant germplasm, wild relatives, landraces, and introgression lines can reveal adaptive traits with breeding relevance that have been maintained under field-relevant selection, including improved Na⁺ exclusion, stronger H⁺ extrusion, better rhizosphere pH buffering, enhanced nutrient acquisition, and reduced growth penalty under alkaline soil conditions (Cao et al. 2020; Zhang et al. 2023; Sun et al. 2023; Cui et al. 2023; Liu et al. 2024). Therefore, integrating genetic diversity with physiological phenotyping is essential for translating alkaline-tolerance mechanisms into breeding targets (Sun et al. 2023; Liu et al. 2024).

In cereals, several recent studies have demonstrated that natural variation can be used to identify alkaline-tolerance loci with breeding potential. Genome-wide association studies in alkali-tolerant cereals identified *Alkaline Tolerance 1* (*AT1*) as a locus associated with high pH tolerance. *AT1* encodes an atypical γ subunit of the heterotrimeric G protein, and its rice (*Oryza sativa*) ortholog is Grain Size 3 (GS3) (Zhang et al. 2023; Sun et al. 2023). Functional analyses show that AT1/GS3 regulates the phosphorylation status of PM aquaporins from the Plasma Membrane Intrinsic Protein 2 (PIP2) subgroup, which affects H_2_O_2_ distribution between the cytoplasm and apoplast and reshapes root redox status under alkaline stress. Loss-of-function or C-terminal truncation of AT1/GS3 increases PIP2 phosphorylation, promotes H_2_O_2_ efflux to the apoplast, reduces intracellular oxidative stress, and improves root growth under high pH. This regulatory role appears conserved across sorghum (*Sorghum bicolor*), rice, maize (*Zea mays*), and wheat (Zhang et al. 2023; Yu et al. 2023; Sun et al. 2023) (Fig. 2** and **Table 1). In maize, natural variation in *Na⁺ Content Under Saline-Alkaline Condition 1* (*ZmNSA1*) reduces shoot Na⁺ accumulation and improves saline-alkaline tolerance, indicating its potential value for breeding tolerant maize varieties (Cao et al. 2020). Together, *AT1*/*GS3* and *ZmNSA1* illustrate how natural variation can identify loci associated with alkaline tolerance and crop adaptation to alkaline soil.

Domestication-associated genetic variation can also affect tolerance to alkaline tolerance of modern cultivars. Tomato tolerance to NaHCO_3_-based alkaline stress was reduced during domestication and improvement. This reduction was associated with changes in the promoter of *SOS3*-*like Ca*^*2*^*⁺-binding Protein 8* (*SlSCaBP8*). Compared with the wild *S. pimpinellifolium* promoter haplotype, the cultivated haplotype showed weaker *SlSCaBP8* expression under NaHCO_3_ treatment (Liu et al. 2024). An introgression line carrying the wild *SlSCaBP8* locus showed enhanced tolerance under NaHCO_3_ treatment, indicating that the wild promoter haplotype is a useful genetic resource for tomato improvement (Liu et al. 2024). These findings identify *SlSCaBP8* as a positive regulator of tomato tolerance to NaHCO_3_-based alkaline stress. Together, these studies suggest that breeding for alkaline soils should prioritize genetic resources from natural variation, domestication, and crop improvement, especially promoter haplotypes and alleles that enhance stress-induced expression without imposing constitutive growth costs (Sun et al. 2023; Liu et al. 2024).

### Alkaline-responsive transcription factor modules

**Fig. 3:**
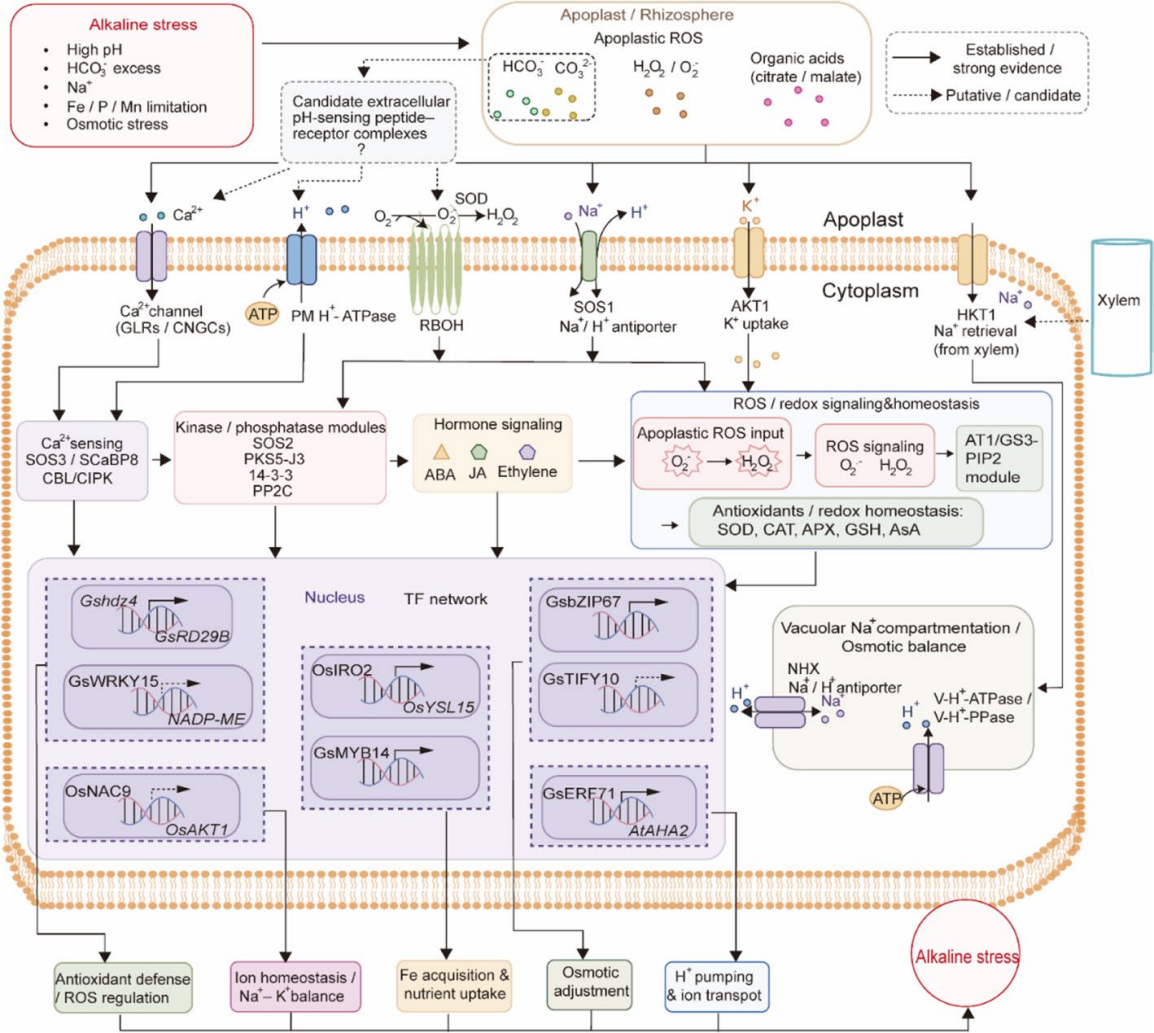
Integrated regulatory model of plant responses to alkaline stress. Alkaline stress induces coordinated responses involving extracellular pH changes, Ca^2^⁺ signaling, reactive oxygen species (ROS)/redox regulation, ion transport, vacuolar Na⁺ compartmentation, transcriptional regulation, and rhizosphere modification. High pH, HCO_3_⁻/CO_3_^2^⁻ accumulation, Na⁺ toxicity, nutrient limitation, and osmotic stress first reshape the apoplast/rhizosphere environment. Candidate extracellular pH-sensing peptide–receptor complexes may contribute to early perception of apoplastic alkalinization, while Ca^2^⁺ influx channels, Ca^2^⁺ sensors, kinase/phosphatase modules, hormone signaling, and ROS/redox pathways transmit early stress signals. Downstream responses include plasma membrane H⁺-ATPase-mediated H⁺ extrusion, Salt Overly Sensitive 1 (SOS1)-mediated Na⁺ efflux, Potassium Channel 1 (AKT1)-mediated K⁺ uptake, High-Affinity K⁺ Transporter 1 (HKT1)-mediated Na⁺ retrieval from the xylem, and Na⁺/H⁺ Exchanger (NHX)/vacuolar H⁺-ATPase/vacuolar H⁺-PPase-dependent vacuolar Na⁺ compartmentation. The nucleus-centered transcription factor (TF) network summarizes representative TF families and genes associated with alkaline-stress responses. The homeodomain-leucine zipper (HD-Zip) module shows that *Glycine soja* Gshdz4 is linked to regulation of the downstream stress-responsive gene *G. soja Responsive to Dehydration 29B* (*GsRD29B*), contributing to antioxidant defense/ROS regulation. The WRKY-domain module shows that the GsWRKY15 is associated with *NADP-Dependent Malic Enzyme* (*NADP-ME*) gene expression in *G. soja*, contributing to ROS/redox regulation. The NAM, ATAF1/2, and CUC2 (NAC) module shows that *Oryza sativa* NAC9 (OsNAC9) is associated with the ion-transport-related gene *OsAKT1*, contributing to ion homeostasis and Na⁺-K⁺ balance in rice. The APETALA2/ethylene-responsive factor (AP2/ERF) module shows that GsERF71 is associated with regulation of *Arabidopsis thaliana plasma membrane H⁺-ATPase 2* (*AtAHA2*), contributing to H⁺ pumping and ion-transport. The Basic Helix-Loop-Helix/Iron-Related Transcription Factor 2 (bHLH/IRO2) module shows that the OsIRO2 is associated with regulation of *O. sativa Yellow Stripe-Like 15* (*OsYSL15*) in *O. sativa*, contributing to Fe acquisition and nutrient uptake. The myeloblastosis (MYB) TF module shows that the *G. max* GmMYB14 is linked to Fe bioavailability and nutrient acquisition in soybean. The basic leucine zipper (bZIP) and TIFY/jasmonate ZIM-domain (TIFY/JAZ) modules show that GsbZIP6*7* and GsTIFY10 are associated with stress-responsive genes in *G. soja*, contributing to osmotic and alkaline-stress adjustment. Genes or gene groups shown in these modules indicate reported downstream, co-induced, or stress-responsive associations, and do not necessarily represent direct binding targets unless experimentally validated

In parallel with transporter- and signaling-related candidate genes, alkaline-responsive genes encoding transcription factors (TFs) provide an additional regulatory layer that coordinates pH regulation, ion transport, hormone signaling, ROS homeostasis, and root development (Fang et al. 2021; Cao et al. 2022; Xiang et al. 2025). Transcriptome studies in halophytes and alkaline-tolerant crops have shown that many TF-encoding genes respond to alkaline stress, but only some display strong and consistent changes in gene expression. In* G. soja* roots treated with NaHCO_3_, RNA-sequencing identified rapid induction of genes encoding TFs from the NAM, ATAF1/2 and CUC2 (NAC), homeodomain-leucine zipper (HD-Zip), APETALA2/ethylene-Responsive factor (AP2/ERF), TIFY/jasmonate ZIM-domain (JAZ), and basic leucine zipper (bZIP) families. Several of these TFs have been functionally tested, and in several cases, their gene expression is more strongly induced under alkaline stress than under neutral salt stress (DuanMu et al. 2015; Cao et al. 2016, 2022; Fang et al. 2021) (Fig. 3** and **Table 1).

Within the HD-Zip and NAC families, *G. soja* provides an alkaline stress-focused module. In *G. soja*, the HD-Zip I-type *Gshdz4* and the NAC-type *GsNAC019* genes are induced by NaHCO_3_ and Na_2_CO_3_ in wild soybean, and ectopic expression of either gene in Arabidopsis improves growth and reduces oxidative damage at high soil pH (Cao et al. 2016, 2017). Promoter and functional analyses indicate that Gshdz4 activates *GsNAC019* promoter and regulates multiple abscisic acid (ABA)- and ROS-related genes, while GsNAC019 modulates ABA sensitivity and gene expression under alkaline stress (Table 1). Together, these TFs form an HD-Zip-NAC cascade that connects alkaline signals to ABA-ROS network (Cao et al. 2017; Fang et al. 2021).

AP2/ERF modules also connect hormone signaling with rhizosphere pH regulation. The Ethylene-Responsive Factor 71 (*GsERF71*) is induced in wild soybean by NaHCO_3_ and potassium bicarbonate (KHCO_3_), and ectopic expression of *GsERF71* in Arabidopsis increases alkaline tolerance. This effect correlates positively with higher PM H⁺-ATPase activity, altered auxin distribution, and improved root growth (Yu et al. 2017; Fang et al. 2021). In grapevine (*Vitis vinifera*), VvERF1B works with MYC2 (VvMYC2) and PM H⁺-ATPase 10 (VvPMA10) to enhance NaHCO_3_ tolerance by stimulating H⁺ extrusion and organic acid exudation, thereby promoting rhizosphere acidification (Xiang et al. 2025) (Table 1).

Jasmonate-related TIFY/JAZ TFs include several alkaline-responsive regulators. In *G. soja*, *GsTIFY10* is induced by NaHCO_3_, and its ectopic expression in Arabidopsis improves alkaline stress tolerance by enhancing ROS scavenging and osmotic adjustment (Zhu et al. 2011). The *GsJAZ2* also improves tolerance when overexpressed in soybean (*G. max*), partly through jasmonate (JA) signaling and compatible solute accumulation under NaHCO_3_ treatment (Zhao et al. 2020). Within the bZIP family, group S members include alkaline-responsive TFs. Ectopic expression of *GsbZIP43* in alfalfa improves the performance of transgenic plants under alkaline stress, suggesting that some bZIPs may act as a node in alkaline stress signaling (Jia et al. 2024; Cao et al. 2022) (Table 1).

Because alkaline soils strongly restrict Fe solubility, Fe acquisition modules are also embedded within the alkaline-stress transcriptome. In rice, the Basic Helix-Loop-Helix (bHLH) transcription factor Iron-Related Transcription Factor 2 (OsIRO2) transcriptionally activates Fe acquisition-related genes, including *Nicotianamine Synthase 1/2* (*OsNAS1/2*), *Nicotianamine Aminotransferase 1* (*OsNAAT1*), *Deoxymugineic Acid Synthase 1* (*OsDMAS1*), *Yellow Stripe-Like 15/2* (*OsYSL15/2*), and the Fe uptake-related *Iron-Regulated Transporter 1* (*OsIRT1*). This regulation promoting phytosiderophore biosynthesis/exudation and Fe uptake under high pH-induced Fe limitation (Li et al. 2016; Ogo et al. 2007) (Fig. 3** and **Table 1). In parallel, ABA-related alkaline-stress datasets highlight a module associated with NAC Transcription Factor 9 (OsNAC9), together with Jacalin-Related Lectin (OsJRL), Peroxisomal Biogenesis Factor 11 (OsPEX11), and the K⁺ channel AKT1-type Potassium Channel (OsAKT1), which is proposed to coordinate Na⁺/K⁺ homeostasis with enhanced antioxidant capacity, partly via peroxisome-linked ROS detoxification (Liu et al. 2019). Finally, in soybean, temporal transcriptome analyses identified MYB Transcription Factor 14 (GmMYB14) as a candidate regulator that activates phenylpropanoid metabolism, providing an additional route to ROS mitigation and potentially improving Fe bioavailability through phenolic chelation under sodic alkaline stress (Zhang et al. 2025b) (Fig. 3 and Table 1).

Overall, alkaline-responsive TFs integrate hormone, redox, and ion/pH pathways and help coordinate rhizosphere acidification and Fe acquisition, Na⁺/K⁺ homeostasis, ROS control, and root growth under alkaline stress. Together with the high-pH-associated regulators identified by genetic studies (e.g., G-protein and Ca^2^⁺-linked components), these modules provide prioritized candidates for functional validation and targets for breeding and genetic engineering to improve crop alkaline tolerance.

## Conclusions and future perspectives

Alkaline stress elevates rhizosphere pH and often co-occurs with high Na⁺ levels and reduced micronutrient availability, which together limit plant growth and productivity. To mitigate these challenges, plants employ coordinated adaptive strategies, including rhizosphere acidification, organic acid exudation, osmotic adjustment, and reinforcement of antioxidant defense systems. Increasing evidence indicates that Ca^2^⁺ and ROS signaling pathways coordinate proton pumping and Na⁺ transport, thereby linking cellular pH and ion homeostasis to root growth and adaptive responses. In contrast, the contribution of specialized structures, such as salt glands or epidermal bladder cells, to alkaline tolerance remains limited and inconsistent across species.

Future research should move from descriptive stress responses toward prioritized, testable mechanisms that connect alkaline-stress perception, root–rhizosphere regulation, molecular control, and field performance. First, early sensing mechanisms should be dissected at cell-type and subcellular resolution. A central hypothesis is that alkaline stress is initially perceived through extracellular pH changes, proton-gradient disturbance, membrane-potential shifts, and secondary Ca^2^⁺/ROS signals in specific root cell types, rather than through a single universal alkaline stress receptor. This can be tested by combining NaHCO_3_/Na_2_CO_3_ treatments with genetically encoded pH, Ca^2^⁺, and ROS sensors, membrane-potential probes, receptor mutants, and live imaging of root hairs, epidermis, cortex, endodermis, and the root apical meristem. Single-cell RNA-sequencing, spatiotemporally enhanced-resolution omics sequencing (Stereo-seq), and cell-type-specific proteomics should then be used to identify which cells first activate pH-sensing modules, PM H⁺-ATPases, SOS/NHX transporters, organic acid metabolism, and stress-responsive TFs. Spatial metabolomics can complement these approaches by mapping organic acids and other metabolites involved in root exudation and local pH buffering under alkaline stress.

Second, future studies should establish causal links between molecular regulators and adaptive traits. A useful working hypothesis is that alkaline tolerance depends on coordinated modules linking PM H⁺-ATPase activation, Na⁺/H⁺ exchange, organic acid secretion, antioxidant regulation, nutrient acquisition, and root architectural plasticity. Time-resolved transcriptomics, chromatin accessibility profiling, ChIP-seq or DAP-seq, phosphoproteomics, metabolomics, and ionomics under matched NaCl, NaHCO_3_, Na_2_CO_3_, and saline-alkali treatments would help distinguish NaHCO_3_/Na_2_CO_3_-specific mechanisms from general salt-stress responses. Gene editing should move beyond knockout validation to promoter editing, allele replacement, and tissue-specific modulation of candidate regulators, thereby testing whether candidate genes directly regulate pH homeostasis, ion transport, redox balance, nutrient uptake, and/or root development.

Third, plant–microbe interactions should be integrated into the same physiological and molecular framework. A testable hypothesis is that beneficial rhizosphere microbes and mycorrhizal fungi enhance alkaline tolerance by reinforcing plant-driven rhizosphere acidification, nutrient mobilization, osmotic adjustment, and hormone/redox regulation. Future experiments should simultaneously quantify rhizosphere pH, root exudates, organic acid release, Fe/P/Mn availability, Na⁺/K⁺ balance, root architecture, microbial community structure and abundance, and host gene expression. Synthetic microbial communities, gnotobiotic systems, split-root designs, and stable-isotope or metabolite-tracing approaches can help determine whether microbial organic acids, extracellular polysaccharides, ACC deaminase activity, and/or mycorrhiza-mediated nutrient uptake directly alter plant alkaline-stress signaling and tolerance.

Finally, mechanisms identified in model systems must be tested for conservation/specificity, field relevance, and trait stacking. Durable alkaline tolerance will likely require combining early pH sensing, H⁺ extrusion, Na⁺ compartmentation, nutrient acquisition, ROS control, and root/rhizosphere remodeling. Comparative studies across halophytes, alkaline-tolerant crops, and sensitive glycophytes should distinguish conserved tolerance modules from species-specific adaptations. Genome editing, marker-assisted selection, and synthetic biology can then be used to stack favorable alleles or regulatory modules, followed by validation under realistic saline-alkali field conditions that include salts, high pH, nutrient limitation, drought episodes, and heterogeneous soil structure. For practical breeding, promising targets include alleles that improve root H⁺ extrusion, Na⁺/K⁺ homeostasis, vacuolar Na⁺ compartmentation, Fe/P acquisition, ROS buffering, and root architectural plasticity. Combining genome-wide association studies, introgression populations, haplotype analysis, gene editing, and field phenotyping will be critical for converting these natural and human selection-based variants into cultivars with stable performance on alkaline soils. Field validation should measure yield, rhizosphere pH buffering, nutrient-use efficiency, ion partitioning, microbiome stability, and trade-offs with growth and reproduction. All these research tasks require a concerted effort from research communities across disciplines.

## Data Availability

Not applicable.

## Abbreviations

ABA: Abscisic acid
ACC: 1-Aminocyclopropane-1-carboxylate
ALMT: Aluminum-activated malate transporter
AMF: Arbuscular mycorrhizal fungi
AMT1: Ammonium transporter 1
AP2/ERF: APETALA2/ethylene-responsive factor
APX: Ascorbate peroxidase
AsA: Ascorbate
*AT1*: *a* *lkaline* *t* *olerance 1*
bZIP: Basic leucine zipper
CAT: Catalase
CBL10: Calcineurin B-like protein 10
DHA: Dehydroascorbate
DHAR: Dehydroascorbate reductase
EBS1: S-ribonuclease binding protein EBS1
EPSs: Extracellular polysaccharides
GmHXK15: Hexokinase 15
GmMAX2a: More axillary growth 2a
GmMYB14: MYB transcription factor 14
GPX: Glutathione peroxidase
GR: Glutathione reductase
GRF6 (14–3-3): General regulatory factor 6 (14–3-3 protein)
*GS3*: *grain size 3*
GsACA1: Autoinhibited Ca^2^⁺-ATPase 1
GsBOR2: Boron efflux transporter 2
GsCBRLK: Calcium/calmodulin-binding receptor-like kinase
GsCHX19.3: Cation/H⁺ Exchanger 19.3
GsCML27: Ca^2^⁺-Binding EF-Hand Protein 27
GSH: Glutathione
Gshdz4: Homeodomain-leucine zipper (HD-Zip) I transcription factor
GsJAZ2: Jasmonate ZIM-domain protein 2
GsMIOX1a: Myo-inositol oxygenase 1a
GsMSRB5a: Methionine sulfoxide reductase B5a
GsNAC019: NAC transcription factor 019
GSSG: Oxidized glutathione
GsSLAH3: Slow anion channel-associated homolog 3
GsSnRK1.1: SNF1-related protein kinase 1.1
GsWRKY15: WRKY transcription factor 15
H_2_O_2_: Hydrogen peroxide
HD-Zip: Homeodomain leucine zipper
HKT: High-affinity K⁺ transporter
J3: DnaJ homolog 3
JA: Jasmonate
JAZ: TIFY/jasmonate ZIM-domain
LcTprxII: Type II peroxiredoxin
MDHAR: Monodehydroascorbate reductase
MdSINA2: Seven in absentia E3 ubiquitin ligase 2
MNS1/2/3: α-Mannosidase 1/2/3
Na_2_CO_3_: Sodium carbonate
NAC: NAM, ATAF1/2 and CUC2
NaHCO_3_: Sodium bicarbonate
NHXs: Na⁺/H⁺ exchangers
O_2_·⁻: Superoxide anion
OsAKT1: Shaker-like K⁺ channel 1
*osa-MIR396c*: *Oryza sativa microRNA396* *c*
OsIRO2: Iron-related transcription factor 2
OsIRT1: Iron-regulated transporter 1
OsJRL: Jacalin-related lectin
OsLOX10: Lipoxygenase 10
OsNAS1: Nicotianamine synthase 1
OsPEX11: Peroxisome biogenesis factor 11 (peroxin 11)
OsPPa6: Soluble inorganic pyrophosphatase 6
OsYSL2: Yellow stripe-like transporter 2
PEPC/PEPCK: Phosphoenolpyruvate carboxylase/phosphoenolpyruvate carboxy kinase
PGPRs: Plant growth-promoting rhizobacteria
PhyB: Phytochrome B
PIN2: PIN-FORMED 2
PIP2: Plasma membrane intrinsic protein 2
PKS5: SOS2-like protein kinase 5
PM H⁺-ATPase: Plasma membrane H⁺-ATPase
POD: Peroxidase
PP2C.D1/8: D-clade type 2C protein phosphatases
RBOHs: Respiratory burst oxidase homologs
ROS: Reactive oxygen species
SCaBP3: SOS3-like calcium-binding protein 3
ScVHA-B: V- H⁺-ATPase subunit B
ScVHA-C: V- H⁺-ATPase subunit C
ScVHA-H: V- H⁺-ATPase subunit H
ScVP: Vacuolar H⁺-pyrophosphatase
SOD: Superoxide dismutase
SOS1: Salt overly sensitive 1
TaCCD1: Ca^2^⁺-dependent calcium ion-binding protein 1
TaSAUR215: Small auxin up RNA 215
TCA: Tricarboxylic acid
TFT4: 14-3–3 Protein TFT4
V-H⁺-ATPase: Vacuolar H⁺-ATPase
V-H⁺-PPase: Vacuolar H⁺-pyrophosphatase
VvERF1B: Ethylene response factor 1B
VvMYC2: Basic helix-loop-helix transcription factor MYC2
VvPMA10: Plasma membrane H⁺-ATPase 10
ZmNSA1: Na⁺ Content Under Saline–Alkaline Condition 1
